# Determinants of gastric cancer screening attendance in Korea: a multi-level analysis

**DOI:** 10.1186/s12885-015-1328-4

**Published:** 2015-05-01

**Authors:** Yunryong Chang, Belong Cho, Ki Young Son, Dong Wook Shin, Hosung Shin, Hyung-Kook Yang, Aesun Shin, Keun-Young Yoo

**Affiliations:** 1 Department of Preventive Medicine, Seoul National University College of Medicine, 103 Daehakro, Jongno-gu, Seoul 110-779 Korea; 2 Department of Family Medicine, Seoul National University College of Medicine, 103 Daehakro, Jongno-gu, Seoul, 110-779 Korea; 3 Department of Social and Humanity in Dentistry, Wonkwang University School of Dentistry, 460 Iksan-dearo, Iksan, 570-749 Korea; 4 Cancer Policy Branch, National Cancer Control Institute, National Cancer Center, 323 Ilsanro Ilsandong-gu, Goyang-si, 410-769 Korea

**Keywords:** Gastric cancer, Screening, Social determinants, Multi-level analysis

## Abstract

**Background:**

We aimed to assess individual and area-level determinants of gastric cancer screening participation.

**Method:**

Data on gastric cancer screening and individual-level characteristics were obtained from the 2007–2009 Fourth Korea National Health and Nutrition Examination Survey. The area-level variables were collected from the 2005 National Population Census, 2008 Korea Medical Association, and 2010 National Health Insurance Corporation. The data were analyzed using multilevel logistic regression models.

**Results:**

The estimated participation rate in gastric cancer screening adhered to the Korea National Cancer Screening Program guidelines was 44.0% among 10,658 individuals aged over 40 years who were included in the analysis. Among the individual-level variables, the highest income quartile, a college or higher education level, living with spouse, having a private health insurance, limited general activity, previous history of gastric or duodenal ulcer, and not currently smoking were associated with a higher participation rate in gastric cancer screening. Urbanization showed a significant negative association with gastric cancer screening attendance among the area-level factors (odds ratio (OR) = 0.73; 95% confidence interval (CI) = 0.57-0.93 for the most urbanized quartile vs. least urbanized quartile).

**Conclusion:**

There are differences in gastric cancer screening attendance according to both individual and regional area characteristics.

## Background

Gastric cancer is one of the most common cancers worldwide, with approximately 989,600 new cases and 738,000 deaths per year, accounting for about eight percent of new cancers [1]. The age-standardized rates of gastric cancer have declined rapidly over recent decades without specific intervention [2,3]. Although the incidence and mortality rate of gastric cancer are decreasing, gastric cancer remains one of the major cancers in Korea [4,5]. According to the Korea Central Cancer Registry Data, gastric cancer was the second most-common incident cancer, comprising 14.8% of all new cancers in 2010, and the third most-common cause of cancer deaths in 2010 [4].

In addition to primary prevention by intervening known risk factors, secondary prevention by utilizing mass screening has also been applied in Korea. As a part of a comprehensive “10-year plan for cancer control”, the National Cancer Screening Program (NCSP) was launched in 1999 [6]. Since then, the NCPS has provided free cancer screening for common cancers, including gastric cancer, to low-income individuals. The NCPS has expanded the target population of the free screening program recently by covering individuals within the lower 50% income bracket of national health insurance and recipients of medical aid [6]. The participation rate of gastric cancer screening has been increasing and, according to the data of the “Korea National Cancer Screening Survey”, the lifetime screening rate of gastric cancer was 77.9%, and the screening rate with recommendation was 70.9% in 2012 [7].

Participation in the screening program has been suggested to be affected by area-level factors, as well as by individual characteristics [8-10]; however, limited studies concerning area-level factors and gastric cancer screening are available. Hence, the present study was aimed to identify the factors associated with gastric cancer screening attendance and to help identify targeted interventions to improve participation in gastric cancer screening. To achieve this goal, associations between individual- and area-level factors and gastric cancer screening attendance were examined using the data from the 2007–2009 Fourth Korea National Health and Nutrition Examination Survey (KNHANES IV).

## Methods

The present study was based on the data from the 2007–2009 KNHANES IV. It is a national household survey that provides comprehensive information on health status, health care utilization, socio-demographics and health behaviors of a nationally representative sample. Subjects were sampled using three-stage probability sampling of areas, survey units, and households. The KNHANES IV consisted of three parts: a health survey, a health examination survey and a nutrition survey. All information was collected by face-to-face interview by a trained interviewer except for information about smoking and alcohol, which were self-reported. All participants agreed to provide written consent to participate in KNHANES.

Among 24,871 individuals who completed the health survey, several exclusion criteria were applied for the current analysis: 12,720 subjects aged less than 40 years were excluded because the National Cancer Screening Program was only provided to subjects 40 years and older. Additional exclusions were made as follows: subjects who had a cancer history (n = 471), non-respondents of gastric cancer screening questions (n = 655), and non-respondents of individual socioeconomic status questions (n = 367). The non-respondents were more likely to be older, men, and not to respond to education and occupation questions than their counterparts. Finally, 10,658 men and women were included in the current study.

The areas defined in the present study were municipal districts (called ‘Si’, ‘Gun’, and ‘Gu’). In 2007–2009, South Korea had 234 municipal districts. The primary survey unit addresses of respondents were linked to areas in the 2010 census data. Overall, a total of 10,658 subjects were nested in 187 areas.

Gastric cancer screening attendance was defined as adherence to NCSP guidelines. The NCSP guidelines recommend gastric cancer screening to population aged 40 and older for every two years by either upper endoscopy or upper GI series. The question for gastric cancer consisted with the screening modality (endoscopy only/upper GI series only/both endoscopy and upper GI series) and the date of the latest screening (within 1 year/between 1–2 years/more than 2 years/never attended to the screening). Individuals who reported never taking a gastric cancer screening examination or those who had undergone examinations more than 2 years prior to the response date were regarded as non-attendants of gastric cancer screening.

Individual explanatory variables included age, gender, household income, education level, marital status, economic activity, health insurance status, self-reported health status, limitation of activity, cigarette smoking status, alcohol drinking habits, presence of depressive symptoms, and gastric or duodenal ulcer history. Household income was calculated by dividing the household monthly income by the square root of the household size (equivalized income) [11]. For health insurance status, we compared individuals with national health insurance (NHI) and those receiving Medicaid, which is a government program for low-income or medically needy individuals. The alcohol use disorder identification test (AUDIT) score was used as an indicator of alcohol use. The AUDIT is composed of 10 questions about alcohol use, and the score is a sum of 10 questions, ranging from 0 to 40. Problem drinking was defined as a score of 12 or higher.

The Composite Deprivation Index (CDI) was used to measure area deprivation [12]. The index is composed of the following domains: unemployment, poverty, housing, labor, and social networks [12]. Urbanization and migration indicated the social cohesion of a region. Urbanization was defined as (100% - the agriculture, fishing, and forestry worker rate (%)). The agriculture, fishing, and forestry worker rate was available from the 2005 Population Census data. The migration rate was also available from the 2005 census data. The number of primary care physicians was based on the data from the 2008 Korean Medical Association’s membership survey and was divided by the 2008 district area (km^2^) from the Land registration statistics of the Ministry of Land, Transport, and Maritime Affairs. The number of gastric cancer screening centers per 10,000 persons was taken from the data of the 2010 National Health Insurance Corporation.

To determine the differences in individual socio-demographic variables according to gastric cancer attendance, Chi-square test was performed. For area-level variables, the mean and standard deviation were calculated.

These data had a multilevel structure comprising 10,658 individuals (at level 1) nested within 187 districts (at level 2). Odds ratios (ORs) and their 95% confidence intervals (CIs) for gastric cancer screening participation were analyzed using multilevel logistic regression models, adjusting for both individual- and area-level variables as fixed effects and allowing for heterogeneity between areas. The area-level random effect of the intercept was assumed to be normally distributed with a mean of zero. First, model 1 was constructed with individual-level variables that were significant at univariate analysis (p < 0.05). Model 2 included variables in model 1 and the area-level variable health care supply, followed by the third model with individual variables and area-level variables, including urbanization, CDI and health care supply (Model 3). Area-level variables were available from 176 to 187 districts among 187 administrative districts. Therefore, the individuals with missing data in area-level variables were excluded in the analysis of Model 2 and Model 3. All the dataset used for this study were publicly accessible, therefore exempted from approval of the Institutional Review Board. All statistical analyses were performed using STATA, version 10.0. Statistical significance was defined as a P value less than 0.05 (two-sided).

## Results

Among 10,658 study subjects, 4,684 (43.95%) individuals participated in gastric cancer screening within the previous 2 years (Table 1). Among non-attendants, 39.5% never participated in a gastric cancer screening and 16.2% underwent examination more than two years prior to the response date. More than half of the study participants were women (n = 6,102 (57.2%)). The gastric cancer screening participation rate was higher among subjects aged 50–59 years and 60–69 years than among those aged 40–49 years and older than 70 years. Gastric cancer screening participants were more likely to have a higher household income, a higher education level, private health insurance, a spouse, a job (economically active) and a gastric or duodenal ulcer history. Current smokers were more likely not to participate in gastric cancer screening. Those who attended the screening were less likely to be medical aid beneficiaries and less likely to have limited general activity. Self-reported health status, depressive symptoms, problem drinking and gender were not related to gastric cancer screening attendance.

**Table 1:** **Characteristics of study population by gastric cancer screening attendance within 2 years (n = 10,658)**

		No	Yes	
		(n = 5974)	(n = 4684)	
		N (%)	N (%)	p-value*
Age (years)	40-49	1854 (31.0)	1391 (29.7)	<0.01
	50-59	1344 (22.5)	1369 (29.2)	
	60-69	1291 (21.6)	1226 (26.2)	
	≥70	1485 (24.9)	698 (14.9)	
Sex	Male	2530 (42.4)	2026 (43.3)	0.35
	Female	3444 (57.7)	2658 (56.8)	
Household income	Quartile 1	1852 (31.0)	1086 (23.2)	<0.01
	Quartile 2	1550 (26.0)	1134 (24.2)	
	Quartile 3	1365 (22.9)	1112 (23.7)	
	Quartile 4	1207 (20.1)	1352 (28.9)	
Educational attainment	Elementary school or uneducated	2716 (45.5)	1822 (38.9)	<0.01
	Middle school	878 (14.7)	763 (16.3)	
	High school	1577 (26.4)	1233 (26.3)	
	University or higher	803 (13.4)	866 (18.5)	
Marital status	Without spouse	1408 (23.6)	703 (15.1)	<0.01
	With spouse	4551 (76.4)	3966 (84.9)	
Economic activity	No	2607 (43.9)	1847 (39.6)	<0.01
	Yes	3337 (56.1)	2823 (60.5)	
NHI vs Medicaid	NHI	5574 (94.2)	4474 (95.9)	<0.01
	Medicaid	341 (5.8)	190 (4.1)	
Private health insurance	No	2699 (46.0)	1563 (33.9)	<0.01
	Yes	3172 (54.0)	3052 (66.1)	
Self-reported health status	Healthy	2268 (38.0)	1764 (37.7)	0.69
	Middle	1927 (32.3)	1548 (33.0)	
	unhealthy	1777 (29.7)	1372 (29.3)	
Limitation of activity	Limited	1543 (55.5)	1130 (44.5)	0.04
	Unlimited	4429 (57.7)	3554 (42.3)	
Smoking				<0.01
	Never	3427 (57.5)	2829 (60.5)	
	Ex-smoker	1216 (20.4)	1069 (22.9)	
	current smoker	1317 (22.1)	775 (16.6)	
Problem drinking (AUDIT) score	0 ~ 11	4922 (83.0)	3862 (83.0)	0.93
	≥12	1005 (17.0)	792 (17.0)	
Depression	No	5105 (85.4)	3987 (85.1)	0.63
	Yes	869 (14.6)	697 (14.9)	
Gastric or duodenal ulcer history	No	5576 (93.3)	4264 (91.0)	<0.01
	Yes	398 (6.7)	420 (9.0)	

*p-value by chi-square test.

The urbanization rate from 187 administrative districts ranged from 64.6% to 99.9% (mean and standard deviation = 94.2% and 8.0%, respectively). The Composite Deprivation Index (CDI) was available from 176 administrative districts, and the average of CDI was 119.88. The average number of primary physicians was 11.48 per km^2^. The average number of gastric cancer screening centers among 179 administrative districts was 0.067 per 1000 persons.

When we compared the characteristics of the study participants who had at least one missing value in any area-level variable (n = 1,045) with those with available information for all area-level variables, individuals with missing values were more likely to be younger, economically active and more likely to have a higher household income, a higher education level, private health insurance. Additionally, these subjects were less likely to be medical beneficiaries and less likely to have limited general activity. Furthermore, they were more likely to participate in gastric cancer screening (data not shown).

Table 2 shows the results of multilevel logistic regression analysis models to test the individual- and area-level factors associated with gastric cancer screening attendance. Model 1 included individual-level variables. Men and women aged 50–59 years or 60–69 years, and individuals in the highest quartile of household income or highest education level were more likely to participate in gastric cancer screening. Living with a spouse, having private insurance, showing limitation of activity, having a gastric or duodenal ulcer history and not being a current-smoker were all associated with participation in gastric cancer screening. However, involvement in economic activity and type of public health insurance were not associated with gastric cancer screening after adjusting other variables.

**Table 2:** **Individual-, area- level factors associated with gastric cancer screening attendance: multilevel logistic regression analysis**

	Model 1	Model 2	Model 3
	OR (95% CI)	OR (95% CI)	OR (95% CI)
**Individual level factors**			
Age (years)			
40-49	1.00	1.00	1.00
50-59	1.48 (1.32-1.66)	1.45 (1.29-1.64)	1.48 (1.31-1.66)
60-69	1.73 (1.51-1.97)	1.69 (1.47-1.94)	1.68 (1.46-1.94)
≥70	1.08 (0.91-1.27)	1.06 (0.89-1.26)	1.05 (0.89-1.25)
Household income			
Quartile 1	1.00	1.00	1.00
Quartile 2	1.04 (0.92-1.17)	1.05 (0.94-1.20)	1.07 (0.94-1.21)
Quartile 3	1.14 (1.00-1.30)	1.15 (1.01-1.32)	1.17 (1.02-1.34)
Quartile 4	1.45 (1.26-1.67)	1.46 (1.27-1.69)	1.49 (1.29-1.72)
Education			
Elementary school or less	1.00	1.00	1.00
Middle school	1.09 (0.96-1.24)	1.12 (0.98-1.27)	1.13 (0.99-1.29)
High school	1.02 (0.90-1.15)	1.01 (0.89-1.15)	1.05 (0.92-1.19)
College or more	1.32 (1.14-1.53)	1.29 (1.11-1.50)	1.34 (1.14-1.56)
Marital status			
Without spouse	1.00	1.00	1.00
With spouse	1.45 (1.29-1.62)	1.45 (1.29-1.63)	1.42 (1.26-1.60)
Economic activity			
No job or no activity	1.00	1.00	1.00
Having a job	1.05 (0.96-1.16)	1.03 (0.94-1.13)	0.99 (0.90-1.09)
NHI vs. Medicaid			
NHI	1.00	1.00	1.00
Medicaid	1.14 (0.93-1.40)	1.16 (0.94-1.43)	1.16 (0.94-1.43)
Private health insurance			
No	1.00	1.00	1.00
Yes	1.45 (1.31-1.61)	1.46 (1.31-1.62)	1.47 (1.32-1.64)
Limitation of activity			
No	1.00	1.00	1.00
Yes	1.12 (1.01-1.24)	1.12 (1.00-1.24)	1.11 (1.00-1.24)
Current smoker			
No	1.00	1.00	1.00
Yes	0.68 (0.62-0.76)	0.70 (0.63-0.78)	0.69 (0.62-0.77)
Gastric or duodenal ulcer history			
No	1.00	1.00	1.00
Yes	1.37 (1.18-1.60)	1.38 (1.18-1.61)	1.35 (1.15-1.57)
**Area level factors**			
Urbanization			
Quartile 1			1.00
Quartile 2			0.89 (0.77-1.03)
Quartile 3			0.79 (0.67-0.94)
Quartile 4			0.73 (0.57-0.93)
CDI			
Quartile 1			1.00
Quartile 2			0.99 (0.85-1.15)
Quartile 3			0.95 (0.81-1.11)
Quartile 4			0.98 (0.82-1.18)
No. of primary physicians per unit area (km^2^)		1.00 (0.99-1.00)	1.00 (1.00-1.00)
No. of gastric cancer screening center per 10,000 persons		1.08 (0.92-1.26)	1.08 (0.91-1.27)

Model 1: included individual level factors those were statistically significant in univariate analysis.

Model 2: variables in Model 1 plus medical service supply (numbers of primary physicians per km2, numbers of gastric cancer screening center per 10,000 persons).

Model 3: variables in Model 1 plus all area level factors (urbanization, CDI, medical service supply).

Model 2 included individual-level variables and the area-level variable medical service supply. Both the number of primary physicians per unit area and number of stomach cancer screening centers per 1000 persons were not significantly related to gastric cancer screening participation.

When additional area-level variables, including urbanization, CDI and health care supply, were added to Model 3, urbanization was the only statistically significant area-level factor. Areas with the most urbanized quartile (odds ratio (OR) = 0.73; 95% confidence interval (CI) = 0.57-0.93) and areas with the second most urbanized quartile (OR = 0.79; 95% CI = 0.67-0.94) had a lower likelihood of a high gastric cancer screening attendance than areas with the lowest urbanized quartile. Considering a model with individual variables and only area deprivation (CDI) among the area-level variables, the OR of gastric cancer screening attendance among individuals living in the most deprived areas compared with those living in the least deprived area was 0.83 (95% CI = 0.71-0.97). However, after adjusting for urbanization, area deprivation (CDI) was not statistically significant in Model 3.

## Discussion

The present nationally representative data showed that the participation rate of gastric cancer screening in the Korean population aged over 40 years was 43.9% in 2007–2009. There were substantial differences in gastric cancer screening participation according to individual socioeconomic- and health-related characteristics. A higher income, a higher education level, having a spouse, having private insurance and having an ulcer history promoted gastric cancer screening, whereas being a current smoker tended not to participate in gastric cancer screening. Limitation of general activity had a marginal association with better participation in gastric cancer screening. In addition, there was a significant regional variance in gastric cancer screening participation. Urbanization and high area deprivation were negatively associated with gastric cancer screening.

Although the gastric cancer screening program is provided for free of charge to NHI members and Medicaid recipients in the lower 50% income bracket, socioeconomic characteristics, particularly household income and education level, were still significant predictors of participation in gastric cancer screening. A lower socioeconomic status as represented by income and education level has been shown to be associated with a reduced likelihood of participation in cancer screening, in combination with age, marital status, health insurance coverage, ethnicity, residential area and other variables, in western countries and Japan [13-15]. Although previous studies in Korea have reported inconsistencies in the relationship between gastric cancer screening and socioeconomic factors [16-18], one recent study using KNHANES III data reported results similar to those in the present study [8]. Income level also affects the possession of private health insurance. A study concerning breast cancer screening including both NCSP and private screening reported that private health insurance was related to higher participation in screening [19].

For other individual-level factors, our results were very similar to those of previous studies on the association between education, marital status, limitation of activity, smoking habit, and history of gastric or duodenal ulcer and participation in gastric cancer screening [8].

The adherence to the screening programs is generally higher in women than men in Korea. However, similar or slightly higher adherence to gastric cancer screening in men than women is consistent with previous study [7,8]. For gastric cancer screening, men are more likely to choose endoscopy as a primary screening modality than women, and the proportion of endoscopy screening is steadily increasing [20].

In the present study, there was significant regional variation for gastric cancer screening after considering individual variables. In model 3, urbanization of area was an important predictor of gastric screening. Living in a more urbanized area showed a reduced likelihood of gastric cancer screening. Although a previous study in Japan reported that living in urban areas was related with lesser participation in gastric cancer screening, the urban variable used in that study was only living in a metropolitan area or not [13]. Urbanization encompasses several entities, including a high migration rate, industrialization, and urban poor. According to a previous study in Sweden, a high migration rate was associated with lower participation in cancer screening [21]. Additionally, in the present study, a higher migration rate was associated with lower participation in gastric cancer screening (data were not shown due to the high correlation between the migration rate and urbanization). High urbanization might cause poor regional cohesion and decreased communication, causing difficulties in information exchange, including cancer screening information [22]. In addition, mobile cancer screening was allowed only in rural areas in Korea and could significantly promote the gastric cancer screening participation rate in those areas.

The most deprived area showed poor participation in gastric cancer screening, a finding that was consistent with previous studies. However, after adjusting for urbanization, the deprivation index was not statistically significant. Due to the high positive correlation between urbanization and CDI, we grouped urbanization-CDI into 4 categories: less urbanized, less deprived areas; less urbanized, more deprived areas; more urbanized, less deprived areas; and more urbanized, more deprived areas. From the analysis using the urbanization-CDI complex variable instead of urbanization and CDI in model 3, no significant difference was found between the results (data not shown).

Our results are generally consistent with previous study which used the KNHANES 2005 and reported positive association between higher educational attainment, highest income and gastric cancer screening rates [8]. Although none of the previous study used multi-level approach for the gastric cancer screening rate, results from the Korean National Cancer Screening Survey suggested socio-economic disparities in both organized and opportunistic gastric cancer screening by education and income levels [23].

The present study has several strengths. First, it was performed using national representative data, allowing generalization of the results. Second, gastric cancer screening attendance included both organized and individual screening attendance. Third, this is the first study to consider both individual- and area-level factors using multilevel analysis for gastric cancer screening attendance in Korea.

However, the current study has several limitations. First, the information on cancer screening and independent variables were based on self-report. Therefore, the present study might not be free from information bias related to self-reporting. Previous studies have demonstrated that self-reporting of cancer screening may overestimate the attendance rate and that the gap between self-reporting and actual attendance depended on individual characteristics, including socioeconomic status [24]. The screening rates of the current study were consistent with the results from the Korean National Cancer Screening Survey, which the lifetime and recommendation screening rates of gastric cancer in 2007–2009 were 55.3-65.1% and 45.6-56.9%, respectively [25]. Second, the KNHANES IV data covered only 187 districts (‘Si’, ‘Gun’, and ‘Gu’) among approximately 250 districts in Korea. Therefore, analysis using sample weighting was not appropriate in the current analysis. Third, the bias related to handling of missing values had to be considered. In the current study, areas with missing values were more likely to be urbanized and have a higher gastric cancer screening participation rate. Therefore, the participation rate in more deprived areas could be underestimated, and the associations between gastric cancer screening attendance and area deprivation could be overestimated in Model 3.

## Conclusion

In conclusion, the present study showed differences in gastric cancer screening attendance according to individual characteristics, including socioeconomic status such as household income, education level, marital status, private health insurance status, and smoking status. Additionally, significant regional variance was found. Higher urbanization was associated with a lower likelihood of gastric cancer screening, but area deprivation was suggestively associated with it. To increase the overall participation rate through the expansion of the current organized screening program, targeted interventions for individuals with a low income, those with a low education level and urban residents should be considered.
